# DAILY CAREGIVING TIME AND SPOUSAL CAREGIVERS’ WELL-BEING: THE MODERATING ROLES OF DAILY MARITAL INTERACTIONS

**DOI:** 10.1093/geroni/igae098.4148

**Published:** 2024-12-31

**Authors:** Iris Yang, Stephanie Wilson

**Affiliations:** SMU, DALLAS, Texas, United States; UNIVERSITY OF ALABAMA AT BIRMINGHAM, Birmingham, Alabama, United States

## Abstract

With more individuals providing informal care (i.e., providing unpaid care to an adult with functional limitations), understanding the impacts of this role is crucial. The literature on the well-being of informal caregivers is divided, with some studies reporting primarily harmful effects and others concluding that caregiving is fundamentally beneficial. This study examined how positive and negative marital interactions moderate the association between caregiving time and caregiver affect in both within- and between-person processes, aiming to clarify the mechanisms behind the varied outcomes observed in caregiver well-being. In the Midlife in the United States Survey (MIDUS), 212 spousal caregivers participated in an 8-day daily diary study, resulting in 1689 days of data. A daily, within-person approach captured the day-to-day fluctuations of caregivers’ experiences and emotional well-being. Multi-level modeling was employed, with covariates including caregiver health, gender, employment status, minority status, education, and time spent caring for people besides a spouse. Results indicated that daily marital strain did not significantly moderate the relationship between daily caregiving time and negative affect, while marital uplifts did, reducing the association between increased caregiving time and negative affect. Conversely, daily marital strain and uplifts did not moderate the association between caregiving time and positive affect. At the between-person level, the proportion of days a caregiver experienced marital strain moderated the association between providing more care and having less positive affect. The results generally support the view that caregiving is predominantly linked with psychological burden while highlighting the potential role of marital uplifts in buffering this association.

